# Mental illness and violent behavior: the role of dissociation

**DOI:** 10.1186/s40479-017-0053-9

**Published:** 2017-01-23

**Authors:** Aliya R. Webermann, Bethany L. Brand

**Affiliations:** 1 University of Maryland Baltimore County, 1000 Hilltop Circle, Math/Psychology Building Rm. 312, Baltimore County, Baltimore, MD 21250 USA; 2 Department of Psychology, Towson University, Towson, 21252 MD USA

**Keywords:** Crime, Criminal behavior, Dissociation, Dissociative disorders, Trauma, Emotion dysregulation, PTSD, Violent crime

## Abstract

**Background:**

The role of mental illness in violent crime is elusive, and there are harmful stereotypes that mentally ill people are frequently violent criminals. Studies find greater psychopathology among violent offenders, especially convicted homicide offenders, and higher rates of violence perpetration and victimization among those with mental illness. Emotion dysregulation may be one way in which mental illness contributes to violent and/or criminal behavior. Although there are many stereotyped portrayals of individuals with dissociative disorders (DDs) being violent, the link between DDs and crime is rarely researched.

**Methods:**

We reviewed the extant literature on DDs and violence and found it is limited to case study reviews. The present study addresses this gap through assessing 6-month criminal justice involvement among 173 individuals with DDs currently in treatment. We investigated whether their criminal behavior is predicted by patient self-reported dissociative, posttraumatic stress disorder and emotion dysregulation symptoms, as well as clinician-reprted depressive disorders and substance use disorder.

**Results:**

Past 6 month criminal justice involvement was notably low: 13% of the patients reported general police contact and 5% reported involvement in a court case, although either of these could have involved the DD individual as a witness, victim or criminal. Only 3.6% were recent criminal witnesses, 3% reported having been charged with an offense, 1.8% were fined, and 0.6% were incarcerated in the past 6 months. No convictions or probations in the prior 6 months were reported. None of the symptoms reliably predicted recent criminal behavior.

**Conclusions:**

In a representative sample of individuals with DDs, recent criminal justice involvement was low, and symptomatology did not predict criminality. We discuss the implications of these findings and future directions for research.

## Background

Stereotypes abound in the media regarding violent behavior and crimes among those with mental illness. One need not look further than popular crime television shows, the latest blockbuster film or news stories on perpetrators of atrocities such as school shootings or terrorist attacks. Researchers have worked to unpack the complex question of what role mental illness plays in violence, if any, especially in light of mass shootings in the United States at Sandy Hook Elementary, Virginia Tech University and Pulse Nightclub, among others. Researchers generally agree that there is some relationship between mental illness and the risk for violence, such that mental illness increases the risk for violence perpetration as well as victimization, but there is less consensus on the specific psychopathology and symptoms that contribute to violence.

### A brief literature review on mental illness and violent behavior

Stereotypes about mental illness and violence are common among the general public. Link, Phelan, Bresnahan, Stueve and Pescosolido [1] presented a large sample (*N* = 1444) with vignettes of people with mental illness, in which no violent behavior or thoughts were described, and inquired how likely it was that the “patient” would be violent. Many participants believed it likely that the hypothetical mentally ill individual would perpetrate violence: 17% of respondents endorsed violence as likely among those with minor interpersonal problems, and 33% and 61% thought violence was likely among people with major depression or schizophrenia, respectively. Individuals with mental illness are frequently aware of others’ negative perceptions of them, which can worsen isolation, negative affect and treatment adherence [2, 3].

Individuals with psychological disorders that are highly stigmatized and misunderstood, such as schizophrenia, borderline personality disorder (BPD) and dissociative identity disorder (DID), often face harmful and inaccurate stereotypes which portray them as dangerous and untreatable menaces who require psychiatric or forensic institutionalization. However, as we will review in this study, it is a myth that individuals with DID are the most likely patients in the mental health system to be violent. Various methodologies have been used to study the link between mental illness and violence including: reporting on the prevalence of mental illness among convicted violent offenders, typically homicide defendants; examining violent behavior and crime among clinical populations; and assessing prevalence of violent behavior and crime among those with mental illness in the general population (see Tables 1, 2, 3, 4 and 5 below for the results of studies utilizing each of these methodologies). Many studies examine only violence perpetration, but some examine victimization as well [4–6] (Table 1).

**Table 1:** Victimization among DD and mixed clinical populations

Study	Data source and timeframe	*N*	Homicide	Severe assault	Any violent crime	IPV (physical/sexual)	Sexual Assault	Diagnoses represented in study
Crisanti et al. (2014) [4]^b^	Adult outpatients at community mental health centers, Hawaii, US, 6-month timeframe	2209	N/A	12.3%	N/A	N/A	Grouped w/severe violence	Other psychotic disorder (26.4%)
								Other severe mental health disorders (18.9%)
								Mood disorders (11.8%)
								Schizophrenia (10.1%)
Teplin et al. (2005) [5]^a^	Adult partial day outpatient, full outpatient and inpatient, Illinois, US, 12-month timeframe	936	N/A	N/A	25.32%	N/A	2.64%	Psychotic/major affective disorder (100%)
Webermann et al. (2014) [6]^b^	94% female outpatients with DID, adult lifetime	275	N/A	N/A	N/A	26.1% (physical)	N/A	DID 98%
								DDNOS 2%

^a^Self-report

^b^Clinician reports

**Table 2:** Violent behavior, homicide and psychopathology among general populations

Study	Data source and timeframe	*N*	Homicide	Severe assault	Any violent crime	Sexual assault	Diagnoses represented in study (among only perpetrators when possible)
Coker et al. (2014) [12]^a/b^	National Comorbidity Survey-Adolescent Supplement, US (ages 13–17), 2001–2004, lifetime timeframe	10,123	N/A	N/A	1.7%^a/b^ (same % for both arrest record and self-report)	N/A	Conduct disorder (20.42%)
							Substance use disorder (19.58%)
							Mood disorder (12.51%)
							ADHD (6.8%)
							Oppositional defiant disorder (4.54%)
							Intermittent explosive disorder (4.29%)
							Eating disorder (3.43%)
							PTSD (2.92%)
							No diagnosis (0.37%)
Elbogen & Johnson (2009) [7]^b^	National Epidemiologic Survey on Alcohol and Related Conditions (NESARC), US, 2001–2003, lifetime timeframe	34,653	N/A	6.78%	17.68%	Grouped w/severe violence	Substance use disorder only (21.41%)
							Mood disorder only (10.47%)
							Mood disorder + substance abuse (8.94%)
							Schizophrenia + substance abuse (0.46%)
							Schizophrenia only (0.40%)
							No diagnosis (58.32%)
							Diagnoses represent total *N*, not just offenders
Federal Bureau of Investigations (FBI)^a^	Uniform Crime Reporting (UCR) Program, FBI, 2014, US, 1 year	318.9 million	.004%	0.23%	0.37%	.03%	N/A
Swanson et al. (1990) [11]^b^	Epidemiological Catchment Area survey, National Institute of Mental Health, US, 1 year timeframe	10,059	N/A		3.66%	N/A	Substance use disorder (41.64%)
							Anxiety disorders (20.13%)
							Mood disorder (9.37%)
							Schizophrenia/schizophreniform disorder (3.92%)
							No diagnosis (44.5%)

^a^Arrests

^b^Self-report

**Table 3:** Psychopathology among convicted homicide offenders

Study	Data source and timeframe	Population	*N*	Diagnoses represented in study
Fazel & Grann (2004) [8]^a^	Sweden state crime register 1988–2011, 14-year timeframe	Convicted homicide offenders, 91.7% male	2005	Substance use disorder (19.7%)
				Personality disorder (11.3%)
				Schizophrenia (8.9%)
				Other psychotic disorder (6.5%)
				Mood disorder (4.8%)
				Anxiety disorder (1.4%)
				Adjustment disorder (2.8%)
				Drug-induced or organic psychosis (2.4%)
				Intellectual disability (0.6%)
				PTSD (0.5%)
				Other axis I disorder (11.9%)
				No diagnosis (8%)
Flynn et al. (2011) [9]^a^	UK and Wales National Confidential Inquiry into Suicide and Homicide by People with Mental Illness (NCI), 1997–2004, 8-year timeframe	Convicted homicide offenders, 90% male	4752	Substance use disorder (8.22%)
				Personality disorder (6.71%)
				Mood disorder (6.32%)
				Schizophrenia (5.8%)
				No diagnosis (68.33%)
Rodway et al. (2014) [13] ^a^	UK and Wales National Confidential Inquiry into Suicide and Homicide by People with Mental Illness (NCI), 2003–2005, 3-year timeframe	Convicted homicide offenders, 90% male	28	Schizophrenia or delusional disorder (50%)
				Personality disorder (21%)
				Substance use disorder (15%)
				Mood disorder (4%)
				No diagnosis (10%)
Simpson et al. (2004) [14] ^a^	Homicide Monitoring Database of the New Zealand Police/Law Enforcement System, New Zealand, 1970–2000, 30-year timeframe	Convicted homicide offenders, 68% male	1498	Schizophrenia (3.67%)
				Other psychotic disorder (1.27%)
				Mood disorder (1.2%)
				Personality disorder (0.73%)
				Substance use disorder (0.67%)
				Neurocognitive disorder (0.60%)
				Intellectual disability (0.60%)
				No diagnosis (91.3%)
Wilcox (1985) [27]^a^	Contra Costa County, California Coroner’s Office (US), 1978–1980, 3-year timeframe	Convicted homicide offenders, gender demographics N/A	71	Antisocial personality disorder (32.4%)
				Substance use disorder (19.7%)
				Schizophrenia (9.85%)
				Neurocognitive disorder (2.82%)
				Adjustment disorder (1.41%)
				Substance-induced psychotic disorder (1.41%)
				Substance-induced psychotic disorder (1.41%)
				Other personality disorder (1.41%)
				No diagnosis (31%)

^a^Arrest records

**Table 4:** Violent behavior and homicide among mixed clinical populations

Study	Data source and timeframe	*N*	Homicide	Severe assault	Any violent crime	Sexual assault/interpersonal violence	Diagnoses represented in study (among only perpetrators when possible)
Bruce et al. (2014) [22]^a/b^	Biomedical Research Centre Case Register Interactive Search tool, UK, 2009, lifetime timeframe	165	N/A	35.2%^b^	46.1%^a^	Sexual assault grouped w/severe violence	Schizophrenia (66.67%)
							Substance use disorder (35.2%)
							Antisocial personality disorder (24.2%)
							Diagnoses represent total *N*, not just offenders
Egeland and Susman- Stillman (1996) [33]^c^	Mothers/survivors of childhood maltreatment, outpatient	24	N/A	N/A	N/A	58.33% childhood maltreatment (dissociation significantly higher among abusive mothers)	N/A
Hodgins et al. (2007) [23] ^a/b^	Adult inpatients in government mental health hospital, UK, lifetime timeframe	205	N/A	33.2%^b^	34.1%^a^	N/A	Schizophrenia (65%)
							Substance use disorder (60%)
							Bipolar disorder I or II (18.5%)
							Schizoaffective disorder (8.78%)
Jordan et al. (1996) [25]^b^	Women entering prison, US, 6–24 month timeframe	805					Substance use disorder (47.4%)
							Borderline personality disorder (28%)
							Antisocial personality disorder (11.9%)
							Major depression (10.8%)
Kaplan et al. (1998) [32]^b^	Adult outpatients in mood disorder clinic, NY, US, lifetime timeframe	122	0%	% N/A but dissociative symptoms significantly/positively correlated with assault	N/A	N/A	DES score above cutoff indicative of potential DD (29%)^d^
Matejkowski et al. (2008) [24]^a^	Indiana Criminal Justice Institute (US), 1990–2002, 12-year timeframe	95	100%	46%	N/A	14%	Mood disorder (74%)
							Schizophrenia (28%)
							Other psychotic disorder (15%)
Quimby and Putnam (1991) [31]^c^	Adult inpatients in state psychiatric hospital, Maine, US, lifetime timeframe	70	N/A	N/A	N/A	% N/A but dissociation significantly/positively correlated w/sexual aggression	DES score above cutoff indicative of potential DD (30%)^e^

*DD* dissociative disorder, *DID* dissociative identity disorder, *N/A* not applicable (not inquired in the study), *DES* Dissociative Experiences Scale

^a^Arrest records

^b^Self-reports

^c^Clinician reports

^d^DES ≥ 25

^e^DES ≥ 30

**Table 5:** Violent behavior and homicide among DD clinical populations

Study	Population	*N*	Violent/homicidal self-states	Severe assault	Any violent behavior	Incarceration	IPV (physical/sexual)	Sexual assault	Childhood maltreatment (physical/sexual)	Homicide
Dell & Eisenhower (1990) [34]^a^	Adolescents, outpatient, 100% DID	11	64%	N/A	55%	N/A	N/A	N/A	N/A	N/A
Kluft (1987) [35]^a^	Mothers, outpatient, 100% DID	75	33.3%	N/A	N/A	N/A	N/A	N/A	16%	N/A
Lewis et al. (1997) [40]^a^	92% male incarcerated homicide defendants, 100% DID	12	N/A	N/A	100%	100%	N/A	N/A	N/A	100%
Loewenstein and Putnam (1990) [36]^a^	100% male in- and outpatients, 100% DID	21	60%	N/A	47%	47%	N/A	13%	N/A	19%
Putnam et al. (1986) [37]^a^	92% female in- and outpatients, 100% DID	100	70% (53% internally homicidal)	N/A	29%	N/A	N/A	20%	N/A	19%
Ross and Norton (1989) [38]^a^	88% female in- and outpatients, 100% DID	236	N/A	N/A	38.3%	38.8%	N/A	N/A	N/A	N/A
Webermann et al. (2014) [6]^a^	94% female outpatients with DID	275	N/A	N/A	N/A	N/A	3.5%	N/A	N/A	N/A

Timeframe is adult lifetime (except for Dell [1990], which is entire lifespan)

*DD* dissociative disorder, *DID* dissociative identity disorder, *N/A* not applicable (not inquired in the study)

^a^Clinician reports

In research on the prevalence of mental illness among violent offenders, multiple studies have found the highest rates of violence among individuals with substance use disorders, rather than schizophrenia, BPD and other psychotic disorders [7–11] (Tables 2 and 3). Rates of substance use disorders (including alcohol use disorders and illicit substance use disorders) among self-reported violent offenders range from 20 to 42% [7, 11, 12] (Table 2). Rates of substance use disorders among convicted homicide offenders are lower but still noteworthy, ranging from 1 to 20% [8, 9, 13, 14] (Table 3).

Other studies have approached the question of how mental illness intersects with violence through examining rates of violent behavior among clinical populations. These studies tend to focus on severe/serious mental illness (SMI), that is, disorders which cause or are associated with serious functional impairment or limitations on major life activities [15]. The majority of studies on violent behavior among SMI patients focus on schizophrenia, although some also include other SMIs such as bipolar disorder and antisocial personality disorder (Table 4). Studies on violent behavior and homicide among individuals with schizophrenia indicate these individuals are at increased risk for both violence perpetration and victimization, but that violence is often predicted by comorbid substance use, medication noncompliance and a recent history of being assaulted [16–18]. Studies on violent behavior among individuals with BPD indicate that emotion dysregulation is a longitudinal mediator of violent behavior and may be a primary mechanism that increases risk for violence in this population [19, 20]. The complex DDs, including DID, have been conceptualized as disorders of emotional dysregulation and are often highly comorbid with BPD [21]. The association of emotion dysregulation with violence in DDs should be further examined.

### Dissociative disorders and violent behavior

Notably missing from almost all studies on the intersection of mental illness and violent crime are individuals with dissociative disorders (DDs), including DID and DD not otherwise specified (DDNOS in DSM-IV)/other specified DD (OSDD in DSM-5). This is true of mixed clinical population studies [22–25], studies on violence and mental illness in the general population [7, 11, 12, 26], as well as forensic studies of convicted violent offenders [8, 9, 13, 14, 27]. Although DID is missing from almost all the research on mental illness and violence, it gets an inordinate amount of focus in films about mental illness, particularly those in the horror and thriller genres such as *Split*, *Psycho, Fight Club* or *Secret Window* which portray people with dissociative self-states as prone to violence including homicide, or within comedies that poke fun at the “outlandishness” of dissociative self-states, such as *Me, Myself and Irene.* Given the paucity of research on violent behavior among individuals with DDs, coupled with the saturation of stereotypic portrayals of DDs in the media, misunderstanding abounds regarding what role dissociation plays in violent behavior, if any.

A few studies have examined dissociative symptoms, rather than DDs, as a predictor of violent interpersonal behavior within mixed clinical populations (Table 4). They typically focus on trait dissociation, that is, chronic and enduring dissociative experiences across multiple contexts [28], compared to state dissociation, e.g., transient, not enduring and time-limited dissociative experiences [29], the latter of which are often anecdotally reported by violent offenders, such as amnesia for a violent episode and violence-related dissociative episodes [30]. Quimby and Putnam [31] found that among adult psychiatric inpatients, trait dissociation was positively correlated with patient sexual aggression via staff reports. Kaplan and colleagues [32] found a positive correlation between trait dissociation and patient-reported general aggression among psychiatric outpatients. Dissociation has also been posited to play a role in the intergenerational transmission of domestic violence: grouping young mothers who were survivors of childhood maltreatment based on whether or not they abused their own children, Egeland and Susman-Stillman [33] found significantly greater trait dissociation among mothers who were abusive as compared to those who were not.

A number of case study reviews, conducted nearly three decades ago, reported high rates of violent behavior among patients with DID, according to reports by their treating clinicians [34–38] (Table 5). These studies were typically conducted with small samples derived from the authoring clinician’s case load, relied on clinician reports rather than patient self-report, utilized adult lifetime reporting timeframes rather than specified time frames (the latter is more typical of current studies on violence and mental illness), and did not attempt to objectively verify violent behavior through criminal records or other official documentation. Many studies inquired about DID patients’ violent and/or homicidal dissociative self-states.^1^ Therapists reported that between 33 and 70% of DID patients had violent self-states [34–37]. At times, aggressive self-states within individuals with DID threaten other self-states, which some patients perceive to be internalized homicidal ideation and/or threats, but if carried out, would result in suicide and not homicide. Some of the studies reviewed above did not distinguish violent self-states who were violent towards the individual themselves versus those who were externally violent toward others [34–36]. Putnam and colleagues [37] make the distinction that while 70% of those with DID had violent or homicidal self-states, 53% of the aggressive self-states were “internally homicidal,” that is, with homicidal ideation toward another self-state. Some DID patients may misperceive these internally aggressive self-states as external violent people, rather than the patient being self-destructive or suicidal [39]. Putnam and colleagues [37] describe internalized homicidal behavior as occurring among 53% of their 100 DID patient sample. Some DID patients may also experience flashbacks of past violence perpetrated by another person against them and mistakenly believe they are perpetrating violence against someone else when in fact they are experiencing an intrusive recollection of the past [39].

Within these aforementioned case studies, clinicians reported that 38–55% of their DID patients had a history of any violent behavior [34, 36–38]. Ross and Norton [38] reported that of 236 DID patients, 29% of the males and 10% of females reported being convicted of a crime, and the same percentage reported a history of incarceration. While the type of conviction and reason for incarceration were not specified, Ross and Norton [38] describe more antisocial behavior among men than women. Loewenstein and Putnam [36] and Putnam and colleagues [37] report high rates of sexual assault perpetration among their DID patient samples. Among an all-male sample, Loewenstein and Putnam [36] reported 13% of patients reported having perpetrated a sexual assault, while in a predominately female sample, Putnam and colleagues [37] reported 20% of patients reported having perpetrated sexual assault. Lewis, Yeager, Swica, Pincus and Lewis [40] reported severe childhood maltreatment and adult psychopathology among 12 DID inmates who were incarcerated for homicide. Two studies found 19% of DID patients had completed homicide [36, 37]. Loewenstein and Putnam [36] attribute this extremely high rate of violent behavior to the childhood maltreatment these patients experienced which increases their risk for aggression and violence, as well as their reliance on an all-male sample, who have higher rates of violence. Alternatively, Putnam and colleagues [37] describe confusion about “personified intraphysic conflicts” among the patients leading to misperceptions about the degree of actual violence among DID patients, as described above.

These numbers are concerning, but they are not consistent with more recent studies of DD patients and clinicians that utilize different sampling techniques and designs. Within the international prospective Treatment of Patients with DD (TOP DD) Network Study, only 2% of clinicians and 4–7% of patients report that DD (including both DID and DDNOS/OSDD) patients perpetrated sexual coercion or sexual assault toward a partner in their adult lifetime [41]. Additionally, rates of perpetration of intimate partner violence were low among DD patients, according to therapists: only 3.5% of DD patients were reported by their TOP DD therapists as having perpetrated physical or sexual abuse toward a partner in their adult lifetime [6].

To date, no studies have examined the variables that might contribute to violence and/or criminal behavior among individuals with DDs. Given the important role that emotion dysregulation has had in predicting violence among individuals with BPD, emotion dysregulation should be examined as a possible contributing factor among individuals with DDs. Dissociative and PTSD symptoms may also be associated with violence or criminal behavior due to the possibility that when highly symptomatic, individuals with DDs may be overwhelmed and unable to manage their symptoms such that they become vulnerable to dyscontrol. Lastly, potential psychological comordities to DDs related to violent behavior within the literature, such as mood and substance use disorders, should be examined as potential explanatorty variables for recent criminal justice involvement.

### The present study

Many questions remain regarding what role mental illness plays in violence. Are mentally ill individuals more likely to perpetrate violence compared to people who do not have mental illness? What psychiatric diagnoses are most highly associated with violent behavior and crime? Are individuals with DDs particularly likely to engage in violent and/or criminal behavior? The present study attempts to provide evidence on violent behavior and crime among individuals with DDs engaged in outpatient treatment.

The purpose of our study was threefold; first, to provide a review of the extant literature on DDs and violent behavior; second, to describe the prevalence of recent criminal justice involvement among a sample of treatment-engaged individuals with DDs; and third, to assess symptomatic predictors of violent behavior and crime among individuals with DDs, including dissociative, emotion dysregulation, posttraumatic stress disorder (PTSD) and depressive symptoms, as well as problematic substance use. We hypothesized that crime rates would be low in our sample of individuals with DDs, with the majority of patients reporting no recent criminal history or involvement with the criminal justice system, unless their involvement was as victims of crime. Additionally, we hypothesized that the aforementioned symptoms (dissociation, emotion dysregulation, PTSD, depression and substance use) would not be significantly associated with recent criminal behavior and justice system involvement.

## Methods

### Procedure

#### Overview and recruitment

Clinician and patient participants were recruited through the Treatment of Patients with Dissociative Disorders (TOP DD) Network study. The TOP DD Network study is a longitudinal educational intervention study of patients with DDs who are diagnosed with either DID or DDNOS/OSDD. Over the course of 1 year, patients and clinicians watched weekly 7–15 min psychoeducational and skills training videos and completed written reflection and behavioral exercises. Additionally, therapist and patient participants completed surveys every 6 months (at baseline, 6, 12, 18 and 24 months) that provided additional clinical and behavioral data.

Clinicians were recruited through listservs for mental health professionals, professional trauma conferences and emails to participated in the first TOP DD study [42, 43]. Clinicians were asked to enroll as a dyad with one DD patient from their caseload. All clinician and patient participants completed a voluntary consent process, and the study was approved by the Towson University Institutional Review Board. Eligibility requirements for patients in the TOP DD Network study included a DD diagnosis (DID, DDNOS or OSDD); being in treatment with their current clinician for at least 3 months prior to starting the study; reading English at an 8^th^ grade level; being willing to continue in individual therapy and complete approximately 2 ½ hours weekly of study activities; and being able to tolerate references to trauma, dissociation and safety struggles.

#### Participants

The total TOP DD Network study included 242 patients who completed baseline measures, presented after the screening measures which verified study eligibility. TOP DD Network study patient participants were majority female (88.6%), Caucasian (82.1%), middle-aged (*Median* = 41), highly-educated (50.9% had at least a college diploma), and primarily resided in the United States (42.3%), although the study recruited internationally with a sizeable portion derived from Norway (27.5%) as well as other countries (30.2%). About half of participants (55.2%) were either in a dating or married relationship. Patients were primarily diagnosed by their therapists as having DID (63.4%). Clinician participants were primarily female (80%) and Caucasian (91.3%). Most reported years of experience as therapists (*Median* = 15), as well as in treating trauma (*Median* = 13), and dissociation (*Median* = 8). Clinicians primarily worked in private practice (81.1%) or in an outpatient clinic or hospital (41.6%).

### Patient measures

#### Criminal justice involvement

DD patients were asked about involvement with the criminal justice system in the last 6 months including contact with the police, charges, convictions, court cases, fines, incarceration, probation, referral to mental health through the criminal justice system, and serving as a criminal witness. Participants could respond yes or no to these questions. Clinicians were not asked about their patients’ recent criminal justice involvement.

#### Trait dissociation

Trait dissociation was measured at baseline by the Dissociative Experiences Scale-II (DES) [28]. DES is a 28-item, 10-point scale (ranging from 0 to 100% of the time) where the participant indicates what percentage of the time a particular dissociative experience occurred within the past month. A meta-analysis by van Ijzendoorn and Schuengel [44] demonstrated test-retest reliability of .78–.93, *α* = .93, and convergent validity of *r* = .67. The measure was scored by adding the item frequency values and dividing by the total number of items, yielding an average summary score for each participant.

#### Emotion dysregulation

Emotional dysregulation was measured at baseline by the Difficulties with Emotion Regulation Scale (DERS) [45]. DERS is a 36-item, 5-point scale (ranging from almost never [0–10% of the time] to almost always [91–100% of the time]) where the participant indicates what percentage of the time a particular difficulty with emotion regulation applies to them. The DERS has six subscales encompassing difficulties with accepting emotions, goal-directed behavior, impulse control, as well as lack of emotional awareness, emotional clarity and emotion regulation strategies. Gratz and Roemer [45] reported *α* > .80 for the six DERS subscales, while Mitsopoulou, Kafetsios, Karademas, Papastefanakis & Simos [46] demonstrated a test-retest reliability ranging from .63 to 81 for the six DERS subscales. The measure was scored by summing the item frequency values.

#### Posttraumatic stress disorder

PTSD symptomatology and severity was measured with PTSD Checklist-Civilian (PCL-C) [47]. The PCL-C is a 17-item, 5-point scale (ranging from not at all to extremely) where a participant indicates how often they have experienced a particular PTSD symptom within the past month. A total score of 50 points is the typical cutoff indicating a possible PTSD diagnosis [48]. Weathers and colleagues [47] reported a test–retest reliability of .96 with a retest interval of 2 to 3 days [47]. The measure was scored by summing all items together.

#### Depression

Depressive disorders were assessed by having clinicians report whether their patient currently had a diagnosis of either dysthymia or major depression (yielding answers of yes or no). Major depressive disorder and persistent depressive disorder (e.g., dysthymia) were assessed as potential predictors of criminal behavior.

#### Substance use

Substance use disorders were assessed by having clinicians report whether their patient currently had a diagnosis of a substance use disorder (differentiated from a substance/medication-induced mental disorder; answers were yes or no.)

### Analyses

Binary logistic regression was utilized to assess symptomatic predictors of recent criminal justice involvement in individuals with DDs. Logistic regression was chosen because it predicts membership for a dichotomous dependent variable (i.e., criminal justice involvement) from multiple independent variables, and is appropriate in cases of unequal group sample sizes. We ran eight separate logistic regressions to assess symptomatic predictors of each of the eight criminal justice involvement variables. We report Nagelkerke *R* squared effect sizes on the significant omnibus models. We adjusted alpha levels to account for multiple hypothesis testing, and the critical *p*-value = 0.0062. The sample size for the logistic regression models was *N* = 125, as variables were used from both clinician and patient surveys, and both pre-baseline screening and baseline surveys, which each contained slightly different sample sizes.

## Results

### Prevalence of recent criminal justice involvement

Among 173 DD patients, 12.7% reported contact with the police within the last 6 months; the reasons for this contact were not queried. The patients reported low rates of recent criminal behavior in the last 6 months (Table 6): 4.8% reported involvement in a court case, although it is unknown what role the patient played in the court proceedings (e.g., witness, victim, alleged criminal); 3.6% were witnesses in a criminal case; 3% reported a legal charge; 1.8% reported a fine (s); 1.2% reported a criminal justice mental health referral; and 0.6% reported having been incarcerated. None of the 173 DD patients reported convictions or probation during the most recent 6 months.

**Table 6:** Six-month patient-reported criminal justice involvement among DD patients in TOP DD network study

Criminal behavior	Total *N*	% endorsing	*χ* ^2^	Nagelkerke *R* squared	DES	DERS	PCL-C	MDD	PDD	SUD
Contact with police	125	12.7%	13.28^*^	0.17	N.S.	N.S.	0.01^*^	N.S.	N.S.	N.S.
Contact with the court system	125	4.8%	26.18^**^	0.59	N/A	N/A	0.01^*^	N.S.	N.S.	0.01^*^
Criminal witness	125	3.6%	5.48	0.17	N/A	N/A	N/A	N/A	N/A	N/A
Charged with an offense	125	3%	12.06	0.45	N/A	N/A	N/A	N/A	N/A	N/A
Fined	125	1.8%	11.65	1.00	N/A	N/A	N/A	N/A	N/A	N/A
Referral to mental health system	125	1.2%	8.68	0.44	N/A	N/A	N/A	N/A	N/A	N/A
Incarceration	125	0.6%	11.65	1.00	N/A	N/A	N/A	N/A	N/A	N/A
Conviction	125	0%	--------	--------	-------	-------	--------	-------	-------	-------
Probation	125	0%	--------	--------	-------	-------	--------	-------	-------	-------

-------- = testing not conducted because no participants endorsed type of criminal justice involvement

N/A = no post-hoc testing conducted

*DD* Dissociative Disorder, *TOP DD Network Study* Treatment of Patients with Dissociative Disorders Study, *DES* Dissociative Experiences Scale, *DERS* Difficulties with Emotion Regulation Scale, *PCL-C* PTSD Checklist-Civilian, *MDD* Major Depressive Disorder, *PDD* Persistent Depressive Disorder (Dysthymia), *SUD* Substance Use Disorder

^*^ *p <* .05

^**^ *p* < .00625 (critical *p*-value)

Regarding the nature of criminal justice involvement, patients had the option of explaining criminal justice involvement they labeled as “other.” Eight individuals elected to complete the “other” open text box, indicating the following: calling the non-emergency police due to loud neighbors; reporting a substance abusing child to the police; reporting criminal offenses; participating in divorce court and domestic violence orders; receiving a traffic ticket; “[meeting] with secret service;” reporting a suspicious vehicle; and being admitted to the hospital with police involvement.

### Symptomatic predictors of criminal justice involvement

Within binary logistic regressions assessing symptomatic predictors of eight types of recent criminal justice involvement, symptomatology significantly predicted recent contact with police, *χ* ^2^ (6) = 13.28, *p* < .05, Nagelkerke *R* ^2^ = 0.17. Post-hoc tests indicated that only PTSD symptoms (via the PCL-C) significantly predicted recent contact with police, *p* < .01. However, after applying the critical *p*-value = 0.0062, neither the omnibus model nor the post-hoc tests remained significant.

Symptomatology also significantly predicted recent contact with the court system, *χ* ^2^ (6) = 26.18, *p* < .001, Nagelkerke *R* ^2^ = 0.59. Post-hoc tests indicated that PTSD symptoms (via the PCL-C) significantly predicted recent contact with the court system, *p* < 01, as well as a substance use disorder diagnosis (via clinician report), *p* < .01. However, after applying the critical *p*-value = 0.0062, the post-hoc tests did not remain significant.

## Discussion

The present study had three aims: first, to provide a review of the extant literature on DDs and violent behavior; second, to describe the prevalence of recent criminal justice involvement among a sample of treatment-engaged individuals with DDs; and third, to assess symptomatic predictors of recent criminal justice involvement within the DD sample.

As we hypothesized, criminal justice involvement among individuals with DDs within the prior 6 months was low, according to patient self-reports. Specifically, patients reported the following in the prior 6 months: 4.8% were involved in a court proceeding, 3.6% were witnesses in a criminal case, 3% had a legal charge, 1.8% received a fine (s), 1.2% received a criminal justice mental health referral, and only 0.6% had been incarcerated. None of the DD patients reported convictions or probation during the past 6 months. This contrasts with prior case study reviews of DID patients in which clinicians reported a history of violent behavior among 29–55% of DID patients, and severely violent crime (e.g., homicide and sexual assault) among upwards of 20% of patients [34, 36–38]. While the prior studies assess lifetime rates, as compared to the present study’s 6-month time frame, and relied on clinician reports rather than patient self-reports, the inconsistencies are instructive. The contrasting results may mean that as sampling and assessment techniques develop, research on individuals with DDs will increasingly suggest they are not as prone to violence or crime as initially thought, as violence towards self may have been conflated with violence towards others. Individuals with DD seem to pose a greater threat to themselves then to anyone else, as reflected in their very high rates of self-injurious behavior and frequent suicide attempts [42, 43, 49].

Additionally, our hypothesis that symptoms of emotion dysregulation, dissociation, PTSD, depression (major depressive disorder and persistent depressive disorder), and substance use disorder were not associated with criminal justice involvement in our sample was supported. Out of eight different types of recent criminal justice involvement, symptoms were able to significantly predict only DD patients’ recent contact with police, as well as recent court involvement, but the former omnibus model did not remain significant after applying the critical alpha which adjusted for Type I error due to multiple hypothesis testing. Regarding recent court involvement, PTSD symptoms and substsance use disorder symptoms significantly predicted recent court involvement, but again, these post-hoc tests did not remain significant after applying the critical alpha. Thus, no symptoms reliably predicted criminal behavior among those with DDs. More importantly, dissociative symptoms did not significantly predict any type of criminal justice involvement among our sample of DD patients. This counters the notion that dissociative symptoms increase risk for criminal and violent behavior. It is also possible that given the high level of dissociation and PTSD among our sample, the strength of the relationships could have been attenuated due to a ceiling effect.

Our study’s major limitations concern selection bias and the nature of data available on patients’ criminal justice involvement. First, our participants are in psychotherapeutic treatment and thus may not be representative of those with DDs who do not present to treatment, nor of those in the criminal justice system who have DDs and dissociation. Additionally, by definition, our sample experience severe and chronic trait dissociation, but some criminal behavior may be more related to state dissociation [29, 30]. Second, our data on patients’ criminal justice system involvement was limited: we did not collect clinician reports of patients’ recent criminal justice involvement, details regarding the nature of patients’ recent criminal justice involvement (i.e., our data on police contact and court cases are ambiguous as to whether they indicate possible criminal behavior or being involved as a witness or victim), nor data on lifetime criminal justice involvement. Many studies on mental illness and violent behavior use lifetime rates, and thus this would facilitate comparisons across studies.

Using patient self-reports of criminal justice involvement in the present study may have provided more accurate responses than only using clinician reports, as it is possible that patients would not report criminal behavior to their clinicians due to social desirability concerns and taboos around criminality, although clinician reports would have been a useful adjunct to patient self-reports. Future studies should review criminal justice records for this population because lifetime memories can be difficult to accurately solicit due to amnesia, and due to the confusion some patients may experience between past and present as well as internal versus external events [39]. Future studies should assess both lifetime and recent criminal justice involvement, utilizing clinician reports and criminal justice records in addition to patient self-reports.

Studies on psychopathology and violent behavior should include DD individuals in their samples. Small forensic studies have assessed DDs in violent offenders [40] but larger epidemiological studies of violent offenders have not included DDs, despite assessing a range of psychopathology among offenders [7–9, 11–14, 26, 27].

## Conclusions

In summary, recent criminal justice involvement among our DD clinical sample is low, according to patient self-reports and is not predicted by dissociative, PTSD or emotion dysregulation symptoms, nor by clinician reported substance abuse disorders or mood disorders. This provides compelling evidence contradicting public and media misconceptions and stereotypes of those with DDs as highly prone to criminality and violence. Public awareness about DDs needs to improve through thoughtful and accurate portrayals of DD, as well as all mental illnesses, in media and literature so that stereotypes and stigma are replaced with understanding and scientifically based knowledge. Enduring stigmas portraying those with mental illness as violent may have considerable negative impacts on their treatment engagement, ability to seek out social support, and overall quality of life [2, 3]. Reductions in stereotypes and stigma will allow those with mental illness to live more comfortably and safely and allow the general public to also be less fearful and more compassionate towards those with DDs and all forms of mental illness.

## Abbreviations

BPD: Borderline personality disorder
DD: Dissociative disorders
DDNOS: Dissociative disorder not otherwise specified
DERS: Difficulties with Emotion Regulation Scale
DES: Dissociative Experiences Scale
DID: Dissociative identity disorder
DSM: Diagnostic and Statistical Manual of Mental Disorders
OSDD: Other specified dissociative disorder
PCL-C: PTSD Checklist-Civilian
PTSD: Posttraumatic stress disorder
SMI: Severe mental illness
TOP DD Network Study: Treatment of Patients with Dissociative Disorders Network Study

## Footnotes

1 Sometimes referred to as personalities, identities or parts.
